# RESOLUTE PET/MRI Attenuation Correction for O-(2-^18^F-fluoroethyl)-L-tyrosine (FET) in Brain Tumor Patients with Metal Implants

**DOI:** 10.3389/fnins.2017.00453

**Published:** 2017-08-11

**Authors:** Claes N. Ladefoged, Flemming L. Andersen, Andreas Kjær, Liselotte Højgaard, Ian Law

**Affiliations:** Department of Clinical Physiology, Nuclear Medicine and PET, Rigshospitalet, University of Copenhagen Copenhagen, Denmark

**Keywords:** PET/MR, attenuation correction, brain tumors, bone density, RESOLUTE

## Abstract

**Aim:** Positron emission tomography (PET) imaging is a useful tool for assisting in correct differentiation of tumor progression from reactive changes, and the radiolabeled amino acid analog tracer O-(2-^18^F-fluoroethyl)-L-tyrosine (FET)-PET is amongst the most frequently used. The FET-PET images need to be quantitatively correct in order to be used clinically, which require accurate attenuation correction (AC) in PET/MRI. The aim of this study was to evaluate the use of the subject-specific MR-derived AC method RESOLUTE in post-operative brain tumor patients.

**Methods:** We analyzed 51 post-operative brain tumor patients (68 examinations, 200 MBq [18F]-FET) investigated in a PET/MRI scanner. MR-AC maps were acquired using: (1) the Dixon water fat separation sequence, (2) the ultra short echo time (UTE) sequences, (3) calculated using our new RESOLUTE methodology, and (4) a same day low-dose CT used as reference “gold standard.” For each subject and each AC method the tumor was delineated by isocontouring tracer uptake above a tumor(T)-to-brain background (B) activity ratio of 1.6. We measured B, tumor mean and maximal activity (T_MEAN_, T_MAX_), biological tumor volume (BTV), and calculated the clinical metrics T_MEAN_/B and T_MAX_/B.

**Results:** When using RESOLUTE 5/68 studies did not meet our predefined acceptance criteria of T_MAX_/B difference to CT-AC < ±0.1 or 5%, T_MEAN_/B < ±0.05 or 5%, and BTV < ±2 mL or 10%. In total, 46/68 studies failed our acceptance criteria using Dixon, and 26/68 using UTE. The 95% limits of agreement for T_MAX_/B was for RESOLUTE (−3%; 4%), Dixon (−9%; 16%), and UTE (−7%; 10%). The absolute error when measuring BTV was 0.7 ± 1.9 mL (N.S) with RESOLUTE, 5.3 ± 10 mL using Dixon, and 1.7 ± 3.7 mL using UTE. RESOLUTE performed best in the identification of the location of peak activity and in brain tumor follow-up monitoring using clinical FET PET metrics.

**Conclusions:** Overall, we found RESOLUTE to be the AC method that most robustly reproduced the CT-AC clinical metrics *per se*, during follow-up, and when interpreted into defined clinical use cut-off criteria and into the patient history. RESOLUTE is especially suitable for brain tumor patients, as these often present with distorted anatomy where other methods based on atlas/template information might fail.

## Introduction

Conventional MRI including T1 weighted imaging after gadolinium contrast is the current method of choice for diagnosis and follow-up of cerebral brain tumors (Galldiks et al., 2015b). However, there are several limitations of clinical importance using MRI. Tumor relapse typically presents as a contrast-enhanced region that can be difficult to distinguish from e.g., post-operative changes or radiation damage (Mullins et al., 2005; Vander Borght et al., 2006; Galldiks et al., 2015a). In gliomas, these post-operative changes are difficult to predict as they occur at different time points after treatment, from within the first 3 months of radiotherapy up to several years after (Galldiks et al., 2015b). Furthermore, because of the known capacity of gliomas to infiltrate surrounding tissue, contrast enhanced MRI does not accurately reflect the actual tumor extension (Watanabe et al., 1992; Buchmann et al., 2016).

Positron emission tomography (PET) imaging is a useful tool for assisting in the correct differentiation of tumor progression from reactive changes. Several tracer options exist to image gliomas, where the radiolabeled amino acid analog tracer O-(2-^18^F-fluoroethyl)-L-tyrosine (FET)-PET is amongst the most frequently used due to its ease of synthesis, high *in vivo* stability, and its fast accumulation into brain tumors independent of blood-brain barrier disruption (Vander Borght et al., 2006; Albert et al., 2016). FET-PET is superior to CT and MRI for estimating the true tumor extent both in low- and high-grade gliomas (Kracht et al., 2004; Vander Borght et al., 2006), and the post-resection PET volume, the biological tumor volume (BTV), is a significant prognostic factor for overall survival in glioblastoma multiforme (Poulsen et al., 2017).

The FET-PET images need to be quantitatively correct in order to be used clinically. To achieve this, we need accurate attenuation correction (AC) in PET/MRI (Vander Borght et al., 2006). Several authors have reported shortcomings of the vendor-provided AC techniques, either due to bone not being accounted for (Andersen et al., 2014), or due to incorrect bone segmentation and density assignment (Dickson et al., 2014). Several methods have been proposed for improving AC of the brain (Ladefoged et al., 2017), with varying degree of detail in regards to bone representation. Some of the best performing methods are atlas/template-based, where a pseudo-CT is build either from a single subject (Izquierdo-Garcia et al., 2014) or by combining CT data from a database of subjects with locally similar MR images (Burgos et al., 2014). This strategy is also adopted in the two simultaneous PET/MRI currently available; The Signa PET/MRI (GE Healthcare, Waukesha WI, USA) uses an atlas-based method for the brain (Wollenweber et al., 2013), and the Biograph mMR (Siemens Healthcare GmbH, Erlangen, Germany) uses an atlas-based method for the whole-body (Paulus et al., 2015; Koesters et al., 2016).

An alternative and more adaptable method is the segmentation-based, where the AC map is constructed from a segmentation of one or multiple MR images, e.g., as it was introduced with the MR-AC method RESOLUTE (Ladefoged et al., 2015).

One limitation in previously published evaluations of the effects of various AC strategies has been the lack of recognized metrics in general clinical use. Instead the evaluations have been based on general neuroscience image processing strategies suited for managing large data sets on a group level. This has delivered measures of e.g., global activity, or in template-based predefined anatomical regions, that, although useful, is not in clinical diagnostic use (Ladefoged et al., 2017). For new PET/MRI AC strategies to be generally accepted in the clinical community it is important that performance is evaluated on a patient-by-patient basis using accepted and well-defined clinical metrics.

In this study we aim to compare the effects of the various AC methods on FET-PET images including RESOLUTE in post-surgery brain tumor patients with metal implants. Neuro-oncology FET-PET imaging is particularly well suited as it primarily relies on a number of well-defined and generally accepted biological metrics and diagnostic cut-offs, and less on a subjective clinical reading.

## Materials and methods

### Patients

**Figure 1:**
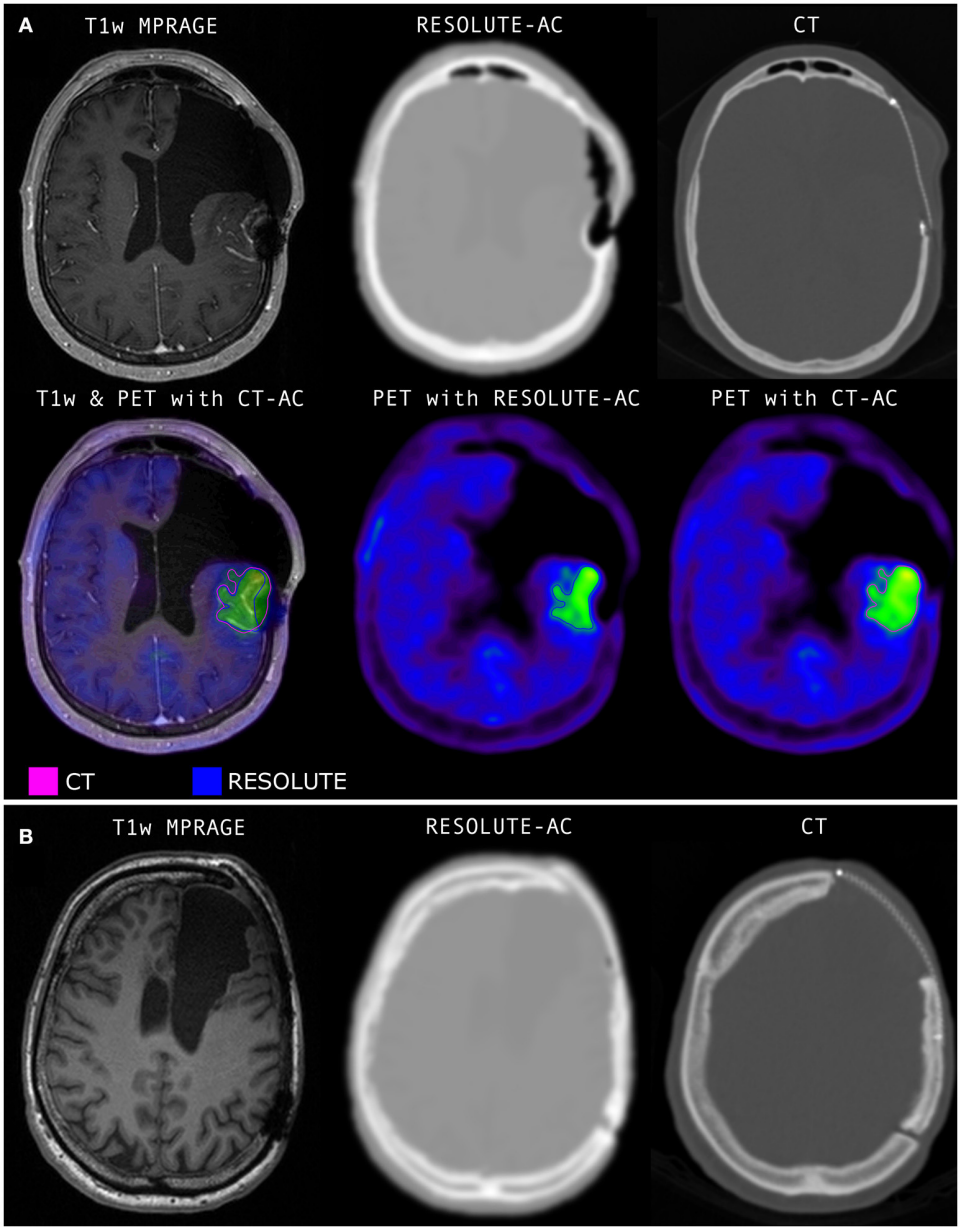
**(A)** The only case in the series where RESOLUTE gave clinical unacceptable results. Not included in the quantitative analyses. The titanium alloy mesh implant give rise to a cigar shaped signal void along the mesh, and a spherical void at it posterior adhesion screw. Bone is build into the lateral frontal subcutaneous tissue swelling and medially into intracranial soft tissue. The FET PET activity distribution shows a significant defect at the posterior signal void. **(B)** A patient with the same type of metal implant but with a minor non-significant signal void in the MR image. Notice, the CTs shown here are before resampling to PET resolution and before blurring is applied to better display the implant. The RESOLUTE attenuation map calculation can be finished in 5 min during the PET acquisition allowing for quality assurance and corrective measures, such as low dose CT of the head for attenuation correction, before the patient leaves the department.

The department archive was screened for patients who underwent surgery for histologically proven glioma or intracerebral metastasis and had simultaneously acquired [^18^F]-FET-PET and MRI using our PET/MRI system (Siemens Biograph mMR, Siemens Healthcare, Erlangen, Germany) (Delso et al., 2011) between February 2013 and April 2016, and 267 FET-PET scans in total were identified. All patients were referred clinically or were part of research protocols, some of which have been published (Henriksen et al., 2016a,b). All investigations were performed on the expressed request and written documentation of the responsible treating clinician, and after obtaining both written and informed consent from the patient at the time of their admission to the hospital for their data to be used in future research. There was no conflict with the Declaration of Helsinki. The regional ethics committee (Scientific Ethics Committee of the Capital Region) has reviewed a synopsis of the protocol, but has waived the need for written informed consent to be obtained from research participants due to the retrospective nature of the study as per national regulations (H-4-2014-FSP). We selected all patients from the cohort with an age above 18 years and a BTV above 1 mL. A total of 52 patients met the inclusion criteria. All had metal implants with titanium alloy clamps in 3–4 trepanation holes for fixation of craniotomized cranial bone flaps (Craniofix®) and/or titanium alloy mesh cranioplasty used in patients with larger cranial calvarial or skull base craniotomy defects. The implants are non-ferromagnetic and MRI-conditional up to 3.0 Tesla, and are known to compromise MR image quality by producing susceptibility artifacts in the near vicinity dependent on magnetic field strength and sequence, usually in the order of 5 mm on T1 MPRAGE. Thus, one repeated examination was removed from group-, but not individual, analysis because of a large signal void from a titanium alloy mesh (Figure 1). One patient was excluded from analysis because of unreliable separation of tumor tissue and physiological skin uptake across AC methods. Fourteen of the included patients had one or more repeated examinations performed (mean difference to baseline: 67 ± 35 days). This resulted in a total of 68 examinations from 51 patients (Table 1). The patients were referred for response assessment during chemotherapy and evaluation of possible tumor recurrence or radiation damage. Primary histology was based on the 2007 World Health Organization (WHO) Classification of Tumors of the Central Nervous System (Louis et al., 2007).

**Table 1:** Characteristics of patients and lesions studied with 18F-FET PET/MRI.

**Characteristic**	**Patients**
Patients	51
^18^F-FET PET scans	68
**Sex**	
Female (%)	20 (39)
Male (%)	31 (61)
Median age (range)	56 (24–82)
**HISTOLOGY AT INITIAL DIAGNOSIS (%)**	
Metastatic carcinoma	2 (4)
Hemangiopericytoma	1 (2)
Metastatic melanoma	1 (2)
Low-grade glioma-unspecified	1 (2)
Astrocytoma grade II	1 (2)
Oligo-astroglioma grade II	3 (8)
Oligodendroglioma grade II	3 (8)
Anaplastic oligodendroglioma grade III	9 (18)
Anaplastic astrocytoma grade III	2 (4)
Glioblastoma multiforme grade IV	28 (55)
**LOCATION OF TUMOR (%)**	
Cortically	26 (51)
Deep-seated	22 (43)
Vermis	3 (8)

### Acquisition of CT

A reference low-dose CT image (120 kVp, 36 mAs, 74 slices, 0.6 × 0.6 × 3 mm^3^ voxels) of the head using a whole-body PET/CT system was used (Biograph TruePoint 40 and Biograph TruePoint 64, Siemens Healthcare) (Jakoby et al., 2009). The CT images were acquired either on the same day as the PET/MRI examination, or at a previous examination with no brain altering surgery in-between.

### Acquisition of MRI

The scan protocol included two vendor-provided AC methods: a two-point DIXON-VIBE AC sequence with repetition time (TR) 2,300 ms, echo time 1 (TE1) 1.23 ms, echo time 2 (TE2) 2.46 ms, flip angle 10 degrees, coronal orientation, 19 s acquisition time, voxel size of 2.6 × 2.6 × 3.12 mm^3^, and a UTE AC sequence with TR/TE1/TE2 = 11.94/0.07/2.46 ms, a flip angle of 10 degrees, axial orientation, 100 s acquisition time, field of view (FOV) of 300 mm^2^, reconstructed on 192 × 192 × 192 matrices (1.6 × 1.6 × 1.6 mm^3^ voxels). The 60 scans performed after October 2013 used the Siemens MR scanner software version VB20P, whereas the 8 scans examined before used software version VB18P, which included a work-in-progress version of the UTE sequence.

### Acquisition of FET-PET

Patients were positioned head first with their arms down on the fully integrated PET/MRI system. Data were acquired 20–40 min after injection of 200 MBq FET over a single bed position of 25.8 cm covering the head and neck. For the purpose of this study, the PET data from the PET/MRI acquisition were reconstructed offline (E7tools, Siemens Medical Solutions, Knoxville, USA) using 3D Ordinary Poisson-Ordered Subset Expectation Maximization (OP-OSEM) with 4 iterations, 21 subsets, zoom 2.5, and 5 mm Gaussian post filtering on 344 × 344 matrices (0.8 × 0.8 × 2.0 mm^3^ voxels) in line with the clinical protocol used at our institution. For all images default random, scatter, and dead time correction were applied.

### Attenuation correction methods

Four methods for AC were applied to the data. First, vendor-provided MR-based attenuation maps, MRAC_DIXON_ and MRAC_UTE_, were derived using the DIXON VIBE sequence (Martinez-Möller et al., 2009) and the UTE MR sequence (Catana et al., 2010), respectively. Next, the MR-AC method RESOLUTE was calculated (Ladefoged et al., 2015). In short, the image-derived method segments the original UTE Echo MR images for air, soft tissue, brain, cerebral spinal fluids, and continuous patient specific bone values. Finally, for each subject, the CT image was co-registered to the T1w image, and was used as our gold standard AC reference following conversion of Hounsfield Units as implemented on the Siemens PET/CT system (Carney et al., 2006): CT-AC.

### Image processing and analysis

The simultaneously acquired FET-PET images were inherently co-registered to the corresponding T1 weighted (T1w) MRI. A 3D “banana” shaped background region (B) of interest (ROI) was delineated in healthy appearing gray and white matter at a level above the insula in the contralateral hemisphere to the tumor. An identical background ROI was used for all 4 AC methods. The BTV of FET-PET was measured using a 3D auto-contour using Mirada XD software (Mirada Medical, Oxford, United Kingdom) defining tumor tissue at a unique threshold above 1.6 of the mean standardized uptake value (SUV) in the background ROI (Floeth et al., 2005) for each AC method separately. Extratumoral areas with high FET uptake, e.g., vascular structures, pineal body and skin, were identified on either the T1w or PET image and removed from evaluation.

The accuracy of the different AC methods in FET-PET was assessed on a patient-by-patient basis using the most commonly used semi-quantitative clinical metrics in the diagnostic workflow. Although, the low-grade gliomas in the study had not received radiotherapy, and other thresholds may be more applicable to metastases (Ceccon et al., 2016), the data from all the patients can be used to simulate the effects of variations in AC techniques on regional activity concentration and clinical metrics. The PET values in the four BTV's were measured as tumor mean (T_MEAN_), and tumor max (T_MAX_), and the ratios T_MEAN_/B and T_MAX_/B were calculated. The ratios were compared across the different MR-AC methods to the ratio measured with the reference CT-AC. These metrics are commonly used as a criterion to identify active tumor tissue from reactive changes. In gliomas post-radiotherapy a T_MAX_/B < 2.0 is often considered reactive tissue (Langen et al., 2011), and a T_MAX_/B > 2.4 is considered indicative of active tumor tissue (Popperl et al., 2006; Galldiks et al., 2015a); while ratios in-between could be in either category. For T_MEAN_/B an optimal cut-off of ~>2.0 combined with time activity curve shape has been found for differentiation between glioma and metastatic recurrence and treatment damage (Galldiks et al., 2015b; Ceccon et al., 2016). However, in clinical practice these cut-off levels should only be considered approximations as the evaluation will also be influenced by a number of other factors including reconstruction parameters, activity morphology, previous and current treatment, structural changes, and prior imaging results.

#### Image metrics and acceptance criteria

Our acceptance criteria were absolute differences of < ±0.05 and 0.1 or 5%, for the T_MEAN_/B and T_MAX_/B ratios, respectively, and ±2 mL or 10% for the BTV. These were based on differences in clinical practice that may be considered clinically relevant in identifying biologically active tumor tissue or treatment related change in activity (Piroth et al., 2011). The mix of both an absolute and relative cut-off reflects that larger absolute change is acceptable in large or very active tumors. There are no test-retest data available for FET-PET imaging.

##### Peak T_MAX_ location

An important FET-PET indication is in biopsy target planning with the identification of the biologically most aggressive component (“hot spots”) in heterogeneous glioma on MRI to optimize the diagnostic accuracy (Messing-Junger et al., 2002; Floeth et al., 2005; Ewelt et al., 2011; Kunz et al., 2011). The biopsy target is usually defined as the peak voxel of T_MAX_ within the tumor. This location was mapped for the different AC reconstructions and compared to the reference. Our criterion was set at < 10 mm, as this corresponds to the approximate size of an average stereotactic biopsy.

##### Biological tumor volume

The BTV has neuro-oncological significance on several levels used both in selected cases as an adjunct in radiotherapy planning (Moller et al., 2016), but also in evaluating treatment response (Albert et al., 2016). Thus, both the BTV value itself and the geometric position and shape needs to be evaluated across AC methods. We analyzed the tumor contours relative to the CT-AC reference using the Jaccard similarity metric, and a measurement of shape deviations, found by thresholding the smoothed tumor difference image. This is in recognition that the clinical impact of a volume change caused by a focal structure is larger than volume change caused by a one-voxel displacement along the tumor contour.

##### Statistical analysis

For each clinical metric, T_MEAN_/B, T_MAX_/B, and BTV, we calculated the mean difference, 95% confidence intervals (CI) and limits of agreement on the log-transformed data, as the data was found to have a log normal distribution. Exponentiation was applied to these results to express the differences as ratios on the original scale and report them as percentage differences:

(1) $$\begin{array}{rcl} \text{CI} & = & 100\cdot \left(e^{d\pm \frac{1.96{SD}_{d}}{\sqrt{n}}}-1\right) \end{array}$$

(2) $$\begin{array}{rcl} \text{Limits of agreement} & = & 100\cdot \left(e^{d\pm 1.96{SD}_{d}}-1\right), \end{array}$$

where d is the mean difference, and *SD*_*d*_ is the standard deviation of the difference on the log scale, where we corrected for repeated measurements from the follow-up examinations (Bland and Altman, 1999). To access the precision, we also calculated the 95% CI for the limits of agreement:

(3) $$\begin{array}{r} \text{C}{\text{I}}_{\text{limits of agreement}}\;=\;100\;\cdot \;\left(e^{d\pm \sqrt{\frac{3SD_{d}^{2}}{n}}}-1\right). \end{array}$$

##### Clinical follow-up analysis

Clinical follow-up defines its own metric. To assess the robustness of the MR-AC methods over time (MR-AC-to-MR-AC) we calculated the within AC modality absolute and percentage changes of BTV, T_MEAN_/B and T_MAX_/B between the baseline and follow-up examinations, respectively, and compared each AC method to the reference CT-AC-to-CT-AC change. Similarly, to simulate the consequences of changing AC methods during the course of clinical follow-up we compared the reference CT-AC-to-CT-AC change with the CT-AC-to-MR-AC change and the MR-AC-to-CT-AC change. For the longitudinal metric we use the same clinical acceptance criteria as for the single time point metric.

A specialist in nuclear medicine (IL) evaluated every tumor delineation visually from the most promising technique (RESOLUTE) compared to CT-AC, and evaluated all metrics that deviated from any defined criteria in relation to the clinical history to identify patients in which this could have impact.

## Results

### Accuracy of attenuation map resolute in the presence of metal implants

**Figure 2:**
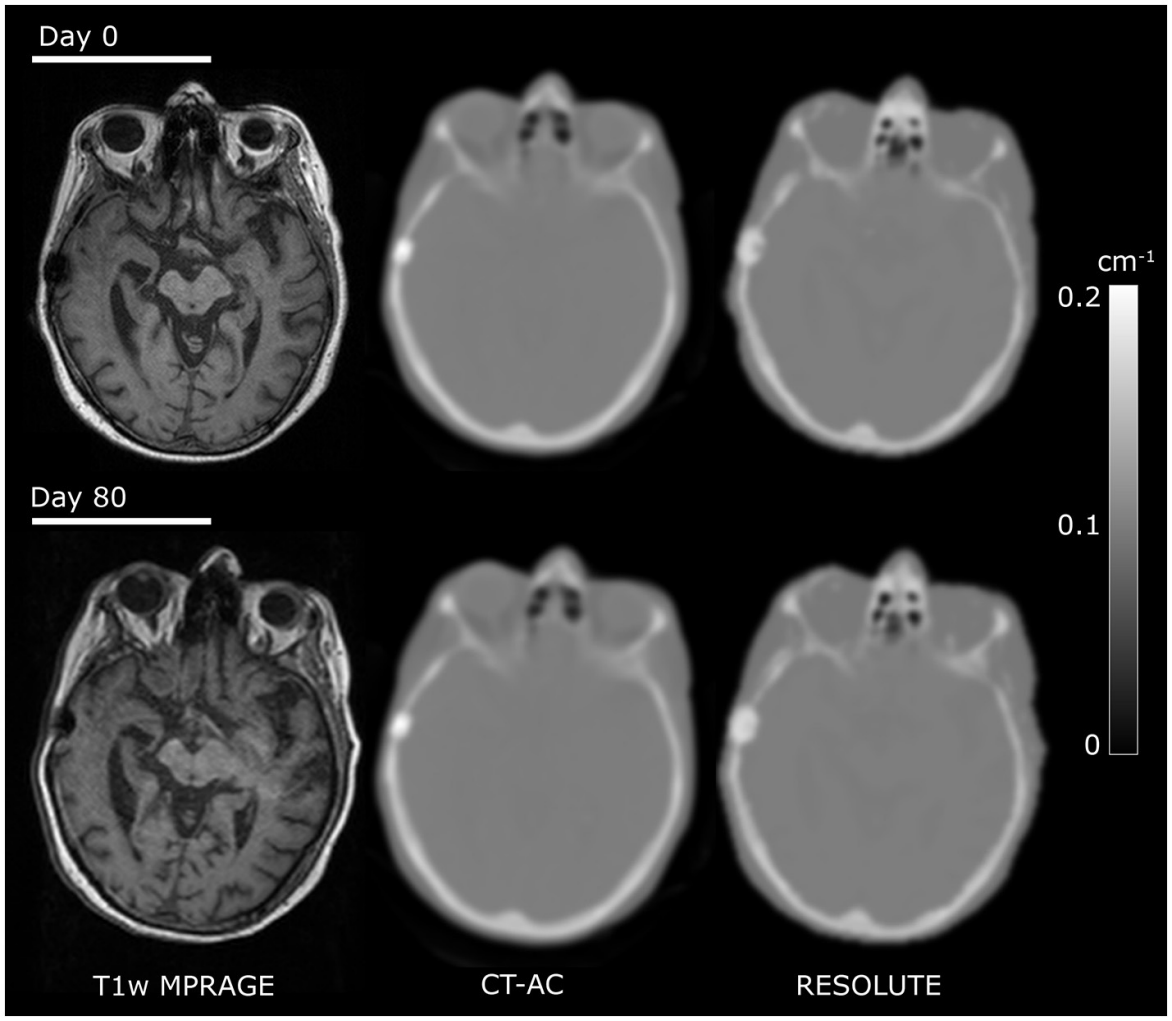
Comparison of CT and RESOLUTE my-maps. Co-registered images from a patient [F, 65 y/o, glioblastoma multiforme (WHO IV)] examined twice within 80 days illustrating good my map repeatability.

RESOLUTE was able to correctly identify and image the inserted titanium implants with one exception, a titanium mesh dominated by signal voids (Figure 1), which was excluded from group analysis. There were 3 scans with an inserted mesh. Visual reading showed that RESOLUTE had the tendency to build up a denser and larger representation of the titanium clamp by 1–3 mm that is apparent from CT-AC (Figure 2).

### Accuracy of pet SUV and diagnostic metrics

**Figure 3:**
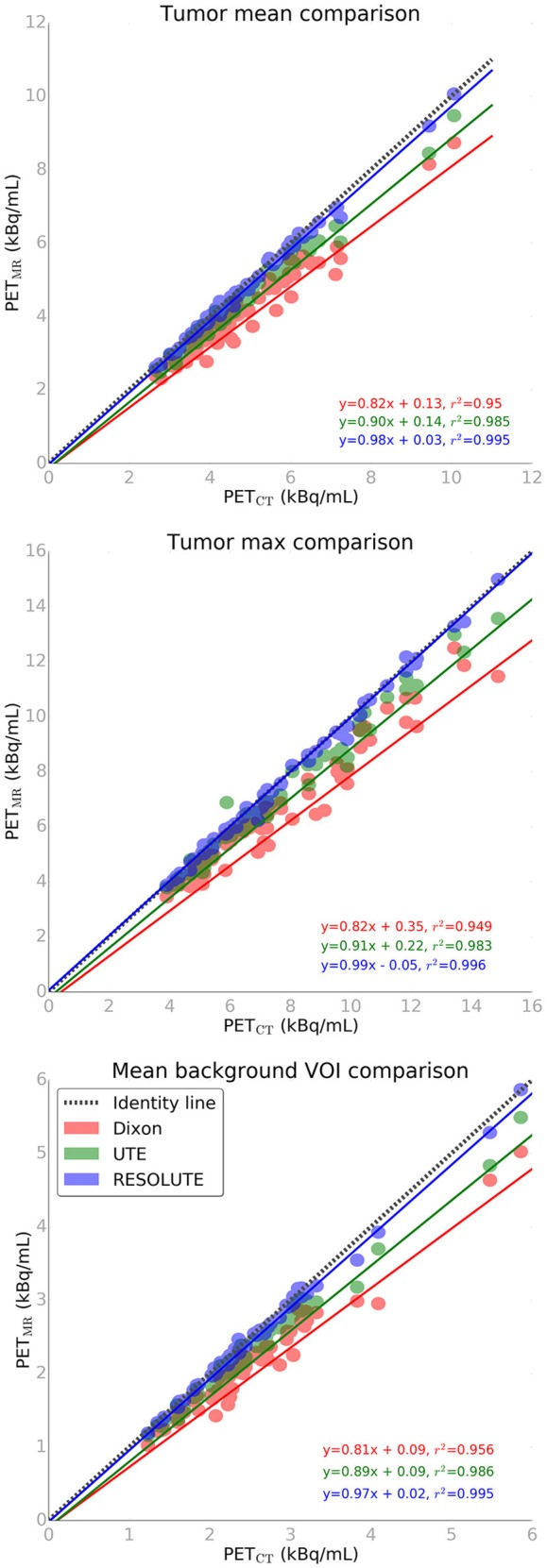
FET-PET tissue activity concentration plot for max tumor (top), mean tumor (middle), and mean background (bottom) values from 3 attenuation correction methods vs. CT reference standard.

The quantitative accuracy of the AC methods can be assessed in Figure 3, and qualitatively in Supplementary Figure 1 for a representative patient. RESOLUTE recovers the tissue activity concentration (kBq/mL) of the T_MAX_ value, and in the T_MEAN_ and background VOI's nearly 100% compared to the reference CT-AC. The relative error of T_MEAN_ was on average −1.9 ± 1.9% (max: −7.6%) using RESOLUTE (Supplementary Table 1). In comparison, the average error for Dixon was −14.9 ± 5.2% (max: −29.3%) and −7.2 ± 3.1% (max: −16.9%) for UTE.

**Figure 4:**
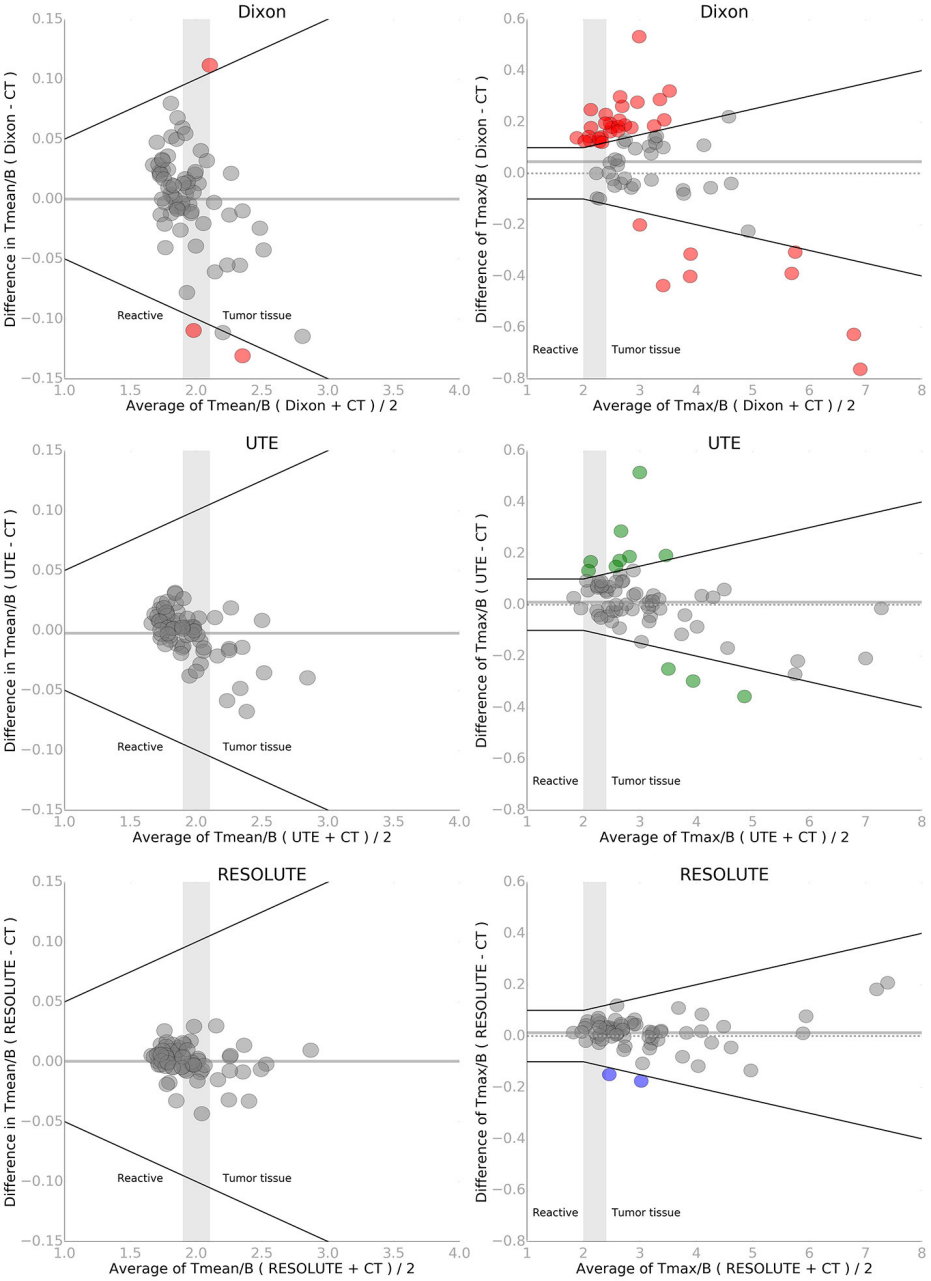
Bland-Altman plot of T_MEAN_/B (left) and T_MAX_/B (right) for each of the three MR-AC methods against the reference standard CT-AC. To simulate the clinical impact of the metrics in evaluating reactive changes vs. tumor recurrence 3 intervals have been labeled along the x-axis. The gray shaded areas define an interval of ambiguity. The black lines indicate the acceptance criteria of T_MEAN_/B of ±0.05 or 5% and T_MAX_/B of ±0.1 or 5%, respectively. Points that exceed the criteria have been colored. Note the difference on the axes. The solid gray line indicates the mean value.

**Figure 5:**
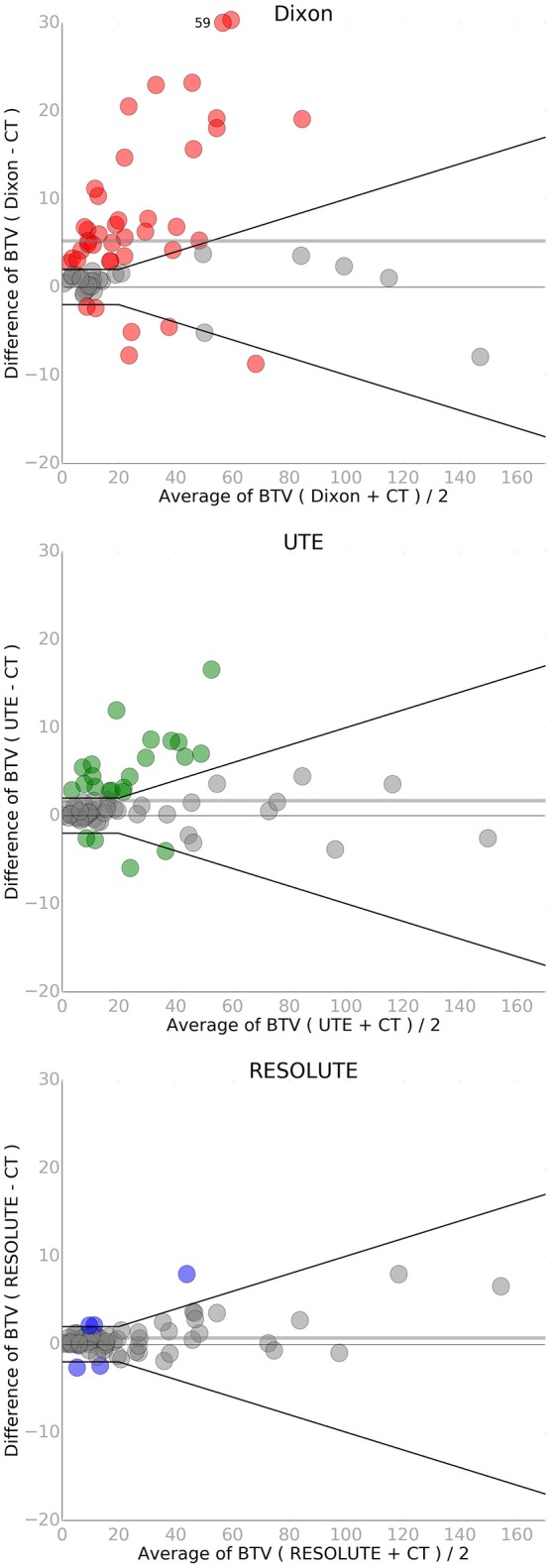
Bland-Altman plot of biological tumor volume (BTV in mL) for each of the three MR-AC methods against the reference standard CT-AC. A single point has been moved (from y = 59 to 30) to fit within the y-axis on the Dixon plot. The black lines indicate the acceptance criteria of BTV of ±2 mL or 10%. Points that exceed the criteria have been colored. The solid gray line indicates the mean value.

When using RESOLUTE 5 out of 68 studies exceed our acceptance criteria of T_MAX_/B difference < ±0.1 or 5%, T_MEAN_/B < ±0.05 or 5%, and BTV < ±2 mL or 10% (Figures 4, 5). In these 5 patients the largest difference for T_MAX_/B was a decrease from 2.53 to 2.39 (6%) and for BTV in an other patient was an increase from 40.0 to 47.9 mL (20%) (Supplementary Figure 2). Characteristically the direction of change in T_MAX_/B and BTV always correlated, and in all cases BTV differences could be assigned to a uniform expansion or erosion of 1–2 voxels around the periphery indicating that the differences were caused by the estimation of activity in the background region. None of the patients changed diagnostic category neither by clinical context, nor by the a priori limits of T_MAX_, because of the differences in FET-PET metrics by RESOLUTE rather than CT-AC. The five studies also exceeded our acceptance criteria when using Dixon or UTE. In total, 46 of the 68 studies exceeded our acceptance criteria for T_MEAN_/B, T_MAX_/B or BTV using Dixon, and 26/68 using UTE.

Comparing the diagnostic measure, T_MAX_/B, with CT-AC between the different AC methods, we found an average difference of 0.014 (N.S.) and a maximal difference of 0.21 using RESOLUTE (Figure 4). The average difference was 0.046 (max: 0.76) using Dixon and 0.010 (max: 0.51) using UTE. The relative difference was smaller with RESOLUTE, with 95% of the patients within 5% of CT-AC (Table 2 and Supplementary Figure 3), compared to within 16% using Dixon and 10% using UTE. A paired *t*-test found a statistical significant difference between the T_MAX_/B measures found with Dixon and CT-AC (*p* < 0.001). The change in AC using Dixon relative to the true classification measured with CT-AC caused six patients to change category from reactive tissue to the equivocal group (*N* = 1) or from the equivocal group to active tumor tissue (*N* = 5). The overall same result is obtained with T_MEAN_/B in terms of improved performance by RESOLUTE, however, all but three patients using Dixon are within our acceptance criteria.

**Table 2:** Summary of the relative %-difference^*^ to the reference CT-AC of each clinical metric for the MR-AC methods.

**Measured parameter values**	**Mean % difference**			**95% lower limits of agreement**	**95% upper limits of agreement**
	**Mean**	**95% CI**	***P***		
**DIXON**					
T_MEAN_/B	0.1	−0.4 to 0.6	0.65	−4.0	4.5
T_MAX_/B	2.6	1.1 to 4.1	<0.001^**^	−9.0	15.7
BTV	32	21 to 43	<0.001^**^	−34	163
**UTE**					
T_MEAN_/B	−0.1	−0.3 to 0.2	0.60	−2.0	1.9
T_MAX_/B	0.9	−0.1 to 1.9	0.08	−7.0	9.5
BTV	11	5 to 17	<0.001^**^	−27	68
**RESOLUTE**					
T_MEAN_/B	0.1	−0.1 to 0.2	0.58	−1.3	1.4
T_MAX_/B	0.4	0.0 to 0.9	0.06	−3.3	4.3
BTV	4	1 to 6	0.01^**^	−16	28

* *Exponentiation was applied to results from analyses on log scale, and results were expressed as percentages*.

** *Indicates a statistical significant (P < 0.05) found by a paired t-test*.

*CI = 95% confidence interval for mean difference*.

*BTV is measured in mL*.

The absolute difference relative to CT when measuring BTV was 0.7 ± 1.9 mL (N.S) using RESOLUTE, where it was 5.3 ± 10 mL using Dixon, and 1.7 ± 3.7 mL using UTE (Figure 5). Also for this metric, the variation was significantly reduced using RESOLUTE. The 95% limits of agreement for this metric is outside the 10% acceptance limit for all MR-AC methods (Table 2), due to the effect of the smaller tumors (<20 mL) on the relative difference measure. This also effects the 95% CI and results in all MR-AC methods being significantly different from CT-AC (*p* < 0.05).

**Figure 6:**
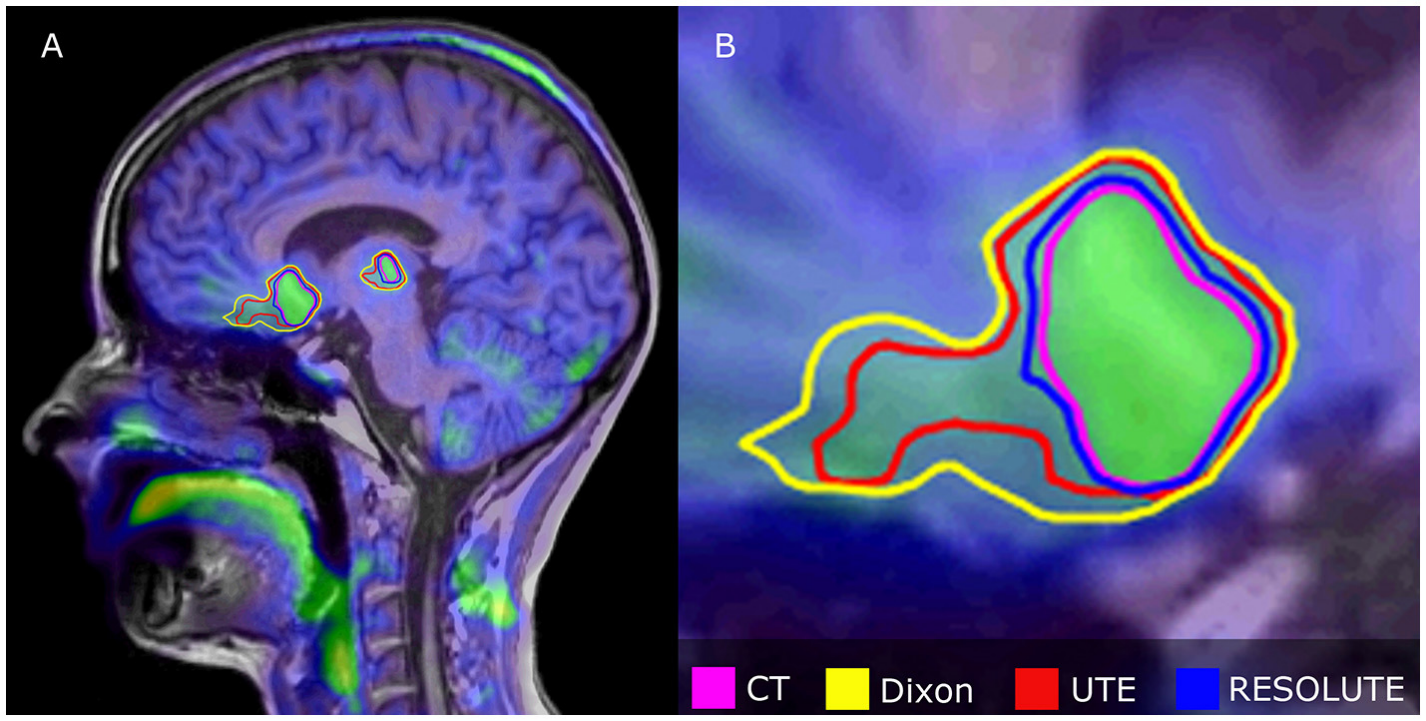
Relative differences in tumor delineation by attenuation correction methods in multifocal Astrocytoma grade II patient overlaid onto FET PET_CT_ fused with T1w MPRAGE. **(A)** Shows sagittal orientation, **(B)** Shows expanded anterior tumor section. RESOLUTE has the best overlap with CT-AC, while Dixon and UTE is significantly overestimated, because of difficulties in defining AC in the skull base and nasal cavity. BTV (mL)/Jaccard Index relative to CT (%) was for CT: 44, Dixon: 75/0.59, UTE: 61/0.72, RESOLUTE: 48/0.89.

The tumor delineation precision was improved, from Jaccard index of 0.70 ± 0.17 using Dixon, to 0.83 ± 0.13 using UTE and 0.90 ± 0.07 using RESOLUTE. The shape deviation analysis found that 37% of the patients had distinct warps in the outline of the BTV of more than 1 mL (average 6 mL) using Dixon, and 11% of the patients using UTE (average 2 mL). These deviations were in the shape of relative expansion toward areas with overestimated tissues in the attenuation map, and relative erosion close to the skull. Using RESOLUTE, none of the patients had tumors with shapes that were significantly different compared to the tumor delineated using CT. An example of the effects of AC method on tumor delineation is illustrated in Figure 6.

The peak location of T_MAX_ used for biopsy guidance was in general agreement across all AC methods. However, in 15% of the patients, the peak location changed position >10 mm compared to CT-AC using Dixon, in 5% when using UTE, and only in 1% using RESOLUTE. In this case the activity peak moved along a low crescent shaped uptake along a resection cavity.

### Follow-up examinations

Fourteen of the patients had a PET/MRI follow-up examination at 5 days to 4 months (average: 66 days) following the first. Pairwise inspection showed that the two RESOLUTE AC maps were well matched (Figure 2). The within AC modality percent change in T_MEAN_/B and T_MAX_/B between the baseline and follow-up examinations are shown for each of the AC methods (MR-AC-to-MR-AC) evaluated relative to CT-AC-to-CT-AC in Supplementary Table 2. The within AC modality changes of T_MEAN_/B and T_MAX_/B were found to be congruent and within criteria for each AC method in almost all patients. In 1 patient the CT-AC-to-CT-AC decrease in T_MAX_/B was 13% (−0.4) while MR-AC-to-MR-AC UTE found an increase of 3% (+0.1). In further 4 patients had absolute differences in T_MAX_/B or T_MEAN_/B, which exceeded our criteria of 5%-points from the reference, with errors above 0.1 using Dixon and UTE, but below 0.2. For BTV 5 patients using Dixon and 3 patients using UTE exceeded our criteria of 10%-points from the reference (Supplementary Table 3). In two patients using Dixon and in two using UTE BTV changes were in increased as opposed to decreased or stable in CT-AC-to-CT-AC, or vice versa. The within AC modality for T_MEAN_/B, T_MAX_/B and BTV follow-up changes were consistent in CT-AC-to-CT-AC and MR-AC-to-MR-AC RESOLUTE.

The between AC modality changes when CT-AC, and not the same MR-AC, was used as baseline for follow-up the deviations in metric were notably more pronounced. We found that 86% of the patients using Dixon, and 50% of patients using UTE, exceeded our criteria of more than either 10%-point in BTV or 5%-point in T_MAX_/B (Supplementary Tables 4, 5). Conversely when using MR-AC as baseline and CT-AC in follow-up there were differences above criteria in 71% of the Dixon patients and 43% of the UTE patients (Supplementary Tables 6, 7). In the most extreme case CT-AC-to-CT-AC yielded a BTV increase of 5% (15.4 to 16.1 mL), which became a 54% increase (15.4 to 23.7 mL) if using CT-AC-to-Dixon-AC, and a decrease of 28% (22.5 to 16.1 mL) in Dixon-AC-to-CT-AC. All RESOLUTE results were within the given criteria except for one patient. Overall, the metric T_MEAN_/B seemed more robust to changes in AC method than T_MAX_/B.

### Discussions

The two most obvious target groups for clinical PET/MRI of the brain is dementia and neuro-oncology patients (Bailey et al., 2015; Barthel et al., 2015; Fink et al., 2015; Werner et al., 2015; Henriksen et al., 2016a,b). Unfortunately, the development, implementation, dissemination, and adaption of PET/MRI has been hampered by the systematic underperformance of an essential correction for accurate PET quantification in the brain (Andersen et al., 2014; Dickson et al., 2014). The clinical consequences of the imperfect AC solutions provided by the scanner vendors have been insufficiently understood and documented leading to reluctance in a full-scale routine clinical adaptation, despite FDA approval. This has lead to a rather long “discovery phase” with a wide range of AC techniques being developed, but usually tested within small studies with a limited number of patients or scans and using metrics that were not in routine clinical use. This has further reduced the confidence in PET/MRI in the clinical and research neuroscience community.

In the present study we have evaluated the effects of 3 MR-AC methods on the clinical metrics used in our institution for FET-PET in a large group of brain tumor patients investigated with PET/MRI scanning.

This category of patients is well suited: (1) It is one of the PET/MRI targets groups; (2) they are particularly challenging in the face of gross anatomical deformation, and MRI susceptibility artifacts from titanium alloy implants, which our patients were retrospectively selected for: (3) it is less dependent on a clinical reading, (4) there are well established clinical semi-quantitative metrics, and (5) the consequences of deviation may be meaningfully interpreted in a clinical context. The latter point is important, as imaging was not followed by biopsy confirmation and we, thus, cannot estimate the diagnostic accuracy of any of the AC methods.

Overall, we found our new RESOLUTE methodology to be the AC method that most robustly reproduced the CT-AC clinical metrics *per se*, during follow-up, and when interpreted into defined clinical use cut-off criteria and into the patient history.

The titanium alloy clamp, that were present in all patients, only generated a limited signal void in the UTE TE images within the width of the skull which meant that a valid attenuation map without artifacts could be calculated in all scans using RESOLUTE (e.g., seen in Figure 2). However, they appeared denser and larger than on CT-AC laterally expanding the BTV for tumors 1–2 mm immediately under a clamp. As present CT-AC techniques do not include a value for metal, CT-AC will classify the clamps as bone and consequently underestimate the attenuation value. Thus, the lateral tumor delineation may be more correct with RESOLUTE than with CT-AC. Unfortunately, this cannot be confirmed with reference to the tumor structure because of the MRI susceptibility artifacts. In a clinical setting this difference will add a smaller volume contribution to the BTV (<1 cm^3^), and can explain some of the larger variabilities in the RESOLUTE-AC BTV measure, but when known this pit-fall can be compensated for in the reading. In 3 scans, patients had a titanium alloy mesh cranioplasty to cover calvarial craniotomy defects, producing unacceptable susceptible artifacts in 1 patient (Figure 1). This patient was subsequently removed from analysis. Furthermore, even though the low-dose CT is obtained at the same day as PET/MRI, we do expect some local registration errors when registering the CT to the MR images. Adding to the misrepresentation of metal, CT must be considered a silver standard here.

The strength of the RESOLUTE methodology is that it is based on an image-derived measurement and is thus able to model abnormal patient anatomy providing that the information is present in the MR images, e.g., in post-operative subcutaneous soft tissue swelling (Supplementary Figure 4). The atlas-based approaches are for dedicated use in a circumscribed, well-defined patient population of which it may be reasonably assumed that the anatomical structures of a patient new to the atlas can be modeled (Burgos et al., 2014; Izquierdo-Garcia et al., 2014). This, unfortunately, seriously restricts the flexibility of PET/MRI as a clinical and research tool and is the most serious flaw in the atlas-based approach. The selection of an atlas-based approach will never satisfy the needs of a diverse clientele for PET/MRI scanning and will always necessitate the implementation of additional AC-methods or atlases for subjects not represented in the primary atlas. Performance of the atlas or template based AC-methods in their present development state can be expected to suffer in patients with titanium implants, cranial defects, and deviant anatomy. Thus, RESOLUTE is a more adaptive tool and a more attractive option from a practical point of view. A further strength of RESOLUTE is processing time. The calculation of an attenuation map can be performed in <5 min. Thus, the quality of the map may be evaluated by the technologist while the patient is still in the scanner. If there are obvious artifacts alternative methods such as CT-AC can be introduced before the patient leaves the department securing an acceptable PET image quality and limiting patient discomfort.

It has previously been shown that both Dixon and UTE systematically underestimates PET tissue activity concentration, which is confirmed in this study (Figure 3). There were no systematic errors when using RESOLUTE. The largest errors when using Dixon and UTE are usually close to bone, as this is not correctly accounted for in either method (Andersen et al., 2014; Dickson et al., 2014), resulting in a radial gradient from the surface of the brain toward the center of the brain. These findings were confirmed also in our patient group (Supplementary Figure 5). The radial gradient is particularly unfortunate using the tracer FET as the tumor boundaries are isocontour-defined dependent on a scan specific threshold from a background area defined in close proximity to the skull. Thus, we found tumor border changes relative to the gradient, expanding in the center, eroding near the skull. More serious than the gradient effects were actual nodular shape deformations of above 1 mL. This was found in 37% of the patients examined using Dixon and in 11% using UTE, and may critically compromise the accuracy and robustness of tumor delineation performed in planning of radiotherapy (Piroth et al., 2012; Gotz and Grosu, 2013; Moller et al., 2016) and of neurosurgical intervention (Klasner et al., 2015; Albert et al., 2016). The largest BTV deviation from CT-AC for RESOLUTE was caused by a 1–2 voxel deviation surrounding the tumor. For two patients, this resulted in an increased BTV compared to CT-AC of 8 mL (+20%) and −3 mL (−40%), respectively. In the first patient this could be attributed to the irregular, large and diffuse tumor borders (Supplementary Figure 2). The difference, however, is sufficiently large to be a concern if considering tumor re-irradiation, where the planning target volume (PTV) consists of margins of only 2–3 mm added to the gross tumor volume (GTV; Grosu et al., 2005; Moller et al., 2016).

Our patient cohort predominantly consisted of FET-PET scans with clear indications of viable tumor tissue of which 29 (43%) had a T_MAX_/B ratio above 3.0. For these patients, even an underestimation of the ratio of up to 0.6 would not change the clinical reading. Thus, only six patients changed clinical category when using Dixon, and none using UTE and RESOLUTE despite the large quantitative errors.

The statistical analysis of the T_MEAN_/B, T_MAX_/B, and BTV assumes log normal distributions. This assumption was confirmed by model control. There were, however, more outlier points than expected when assuming normal distributions, which could result in the limits of agreement being slightly too small.

The results of the follow-up analysis showed that for the majority of the patients a similar conclusion of treatment response regardless of the attenuation method employed would be reached (Supplementary Tables 2, 3). However, the conclusion based on BTV is misleading for two patients using Dixon and two patients using UTE. One of the patients has a decrease in BTV after treatment of 10%, where UTE instead finds an increase of 50%. Such errors could potentially result in the termination of an effective treatment. In contrast, there were no such outliers when using RESOLUTE, but a larger follow-up study should further confirm this. The results of analysis where the AC method was changed in either the baseline or the follow-up examination, and CT-AC was used in the other, illustrated the robustness of RESOLUTE, and indicated that RESOLUTE can potentially replace the CT-AC even in longitudinal studies.

## Conclusion

The present study performed in a large group of post-surgical brain tumor patients using simultaneously acquired FET PET/MRI imaging revealed that our new brain MR-AC method RESOLUTE is able to robustly produce attenuation maps even when titanium alloy clamps are present. The MR images were unpredictable in the case of titanium alloy mesh implants, and visual inspection of the attenuation maps to detect signal voids is still required. The clinical metrics was within acceptable limits of the reference CT-AC, and is an improvement over the vendor-provided Dixon and UTE methods. RESOLUTE is especially suitable for brain tumor patients, as these often present with abnormal anatomy where other methods based on atlas or template information might fail. In our unit we are sufficiently confident in these results to have abandoned the routine use of CT-AC and adapted RESOLUTE-AC for routine clinical PET/MRI FET brain imaging in the adult patients.

## Ethics statement

The data in this study were analyzed retrospectively and based on patients referred for medically necessary clinical FET PET/MRI scanning. All investigations were performed on the expressed request and written documentation of the responsible treating clinician and after obtaining the consent of the patient. There was no conflict with the Declaration of Helsinki. The regional ethics committee (Scientific Ethics Committee of the Capital Region) was asked, but wavered the right to require of further written consent from the patients (H-4-2014-FSP).

## Author contributions

CL designed the method, did the data analysis and prepared the manuscript. FA and IL designed the method, aided in data analysis, revised and approved the manuscript. AK and LH aided in data acquisition, revised and approved the manuscript.

### Conflict of interest statement

The authors declare that the research was conducted in the absence of any commercial or financial relationships that could be construed as a potential conflict of interest.
