# Supplementary material for: RESOLUTE PET/MRI Attenuation Correction for O-(2-18F-fluoroethyl)-L-tyrosine (FET) in Brain Tumor Patients with Metal Implants
Source: Front Neurosci. 2017 Aug 11;11:453. doi: 10.3389/fnins.2017.00453 (PMC5554515; doi:10.3389/fnins.2017.00453)
Supplement: Supplementary file 1 [file Table1.DOCX]

Supplementary Material

RESOLUTE PET/MRI attenuation correction using
O-(2-18F-fluoroethyl)-L-tyrosine (FET) in brain tumor patients

Claes N. Ladefoged, Flemming L. Andersen, Andreas Kjær, Liselotte Højgaard, and Ian Law.

Department of Clinical Physiology, Nuclear Medicine and PET, Rigshospitalet, University of Copenhagen, Denmark

*** Correspondence:** Flemming Littrup Andersen: flemming.andersen@regionh.dk

# Supplementary Data

Supplementary Table 1: Summary of the relative % difference in tissue activity to the reference CT-AC of T_MEAN_ and T_MAX_ for the MR-AC methods plotted in Fig. 3. Each entry displays mean (95% lower limits of agreement; upper limits of agreement).

|  | **T_MEAN_ percent difference** | **T_MAX_ percent difference** |
| --- | --- | --- |
| Dixon | -14.9% (-25.2;-4.7)% | -12.8% (-25.1;-0.4)% |
| UTE | -7.2% (-13.3;-1.1)% | -6.3% (-14.9;2.4)% |
| RESOLUTE | -1.9% (-5.6;1.9)% | -1.5% (-5.8;2.9)% |
