# Supplementary material for: RESOLUTE PET/MRI Attenuation Correction for O-(2-18F-fluoroethyl)-L-tyrosine (FET) in Brain Tumor Patients with Metal Implants
Source: Front Neurosci. 2017 Aug 11;11:453. doi: 10.3389/fnins.2017.00453 (PMC5554515; doi:10.3389/fnins.2017.00453)
Supplement: Supplementary file 6 [file Table6.DOCX]

Supplementary Material

RESOLUTE PET/MRI attenuation correction using
O-(2-18F-fluoroethyl)-L-tyrosine (FET) in brain tumor patients

Claes N. Ladefoged, Flemming L. Andersen, Andreas Kjær, Liselotte Højgaard, and Ian Law.

Department of Clinical Physiology, Nuclear Medicine and PET, Rigshospitalet, University of Copenhagen, Denmark

*** Correspondence:** Flemming Littrup Andersen: flemming.andersen@regionh.dk

# Supplementary Data

**Supplementary Table 6:** Quantitative differences in percent and absolute change between baseline and follow-up for the CT-AC-to-CT-AC versus each of the MR-AC-to-CT-AC methods. The bolded entries indicate T_MAX_/B and T_MEAN_/B changes of at least 5%-points from the reference.

| **T_MAX_/B** | **CT- to-CT** | **Dixon-to-CT** | **UTE-to-CT** | **RESOLUTE-to-CT** |
| --- | --- | --- | --- | --- |
| 1 | -20.8% (-0.53) | **-28.2% (-0.79)** | -25.8% (-0.71) | -21.7% (-0.56) |
| 2 | 0.8% (0.02) | **-9.9% (-0.28)** | **-4.8% (-0.13)** | 1.0% (0.02) |
| 3 | -28.9% (-0.92) | -30.6% (-0.99) | -29.2% (-0.93) | -29.3% (-0.93) |
| 4 | 2.6% (0.18) | **12.5% (0.81)** | 5.7% (0.39) | -0.0% (-0.0) |
| 5 | -4.0% (-0.1) | **-11.3% (-0.29)** | -6.1% (-0.15) | -5.3% (-0.13) |
| 6 | 0.4% (0.01) | **-7.8% (-0.27)** | 1.9% (0.06) | 1.4% (0.04) |
| 7 | -8.8% (-0.24) | -7.5% (-0.2) | -5.6% (-0.15) | -9.6% (-0.26) |
| 8 | -9.6% (-0.21) | -14.4% (-0.34) | -12.2% (-0.28) | -12.0% (-0.27) |
| 9 | -12.0% (-0.31) | -11.2% (-0.28) | -13.9% (-0.36) | -14.2% (-0.37) |
| 10 | -0.4% (-0.02) | **5.1% (0.29)** | 3.5% (0.2) | -1.6% (-0.1) |
| 11 | -16.3% (-0.43) | **-21.9% (-0.62)** | -19.1% (-0.52) | -17.8% (-0.48) |
| 12 | -2.1% (-0.05) | 2.3% (0.05) | 0.2% (0.0) | -1.4% (-0.03) |
| 13 | -26.8% (-0.72) | -30.2% (-0.85) | -26.3% (-0.7) | -27.5% (-0.75) |
| 14 | -13.3% (-0.42) | -18.0% (-0.6) | -12.9% (-0.41) | -13.2% (-0.42) |
|  |  |  |  |  |
| **T_MEAN_/B** | **CT-to-CT** | **Dixon-to-CT** | **UTE-to-CT** | **RESOLUTE-to-CT** |
| 1 | -5.5% (-0.1) | -4.5% (-0.1) | -4.6% (-0.11) | -5.2% (-0.11) |
| 2 | 0.5% (0.01) | 0.5% (-0.06) | 1.7% (-0.02) | 0.8% (0.01) |
| 3 | -13.8% (-0.28) | -12.6% (-0.29) | -14.1% (-0.27) | -14.0% (-0.28) |
| 4 | 21.6% (0.51) | 16.7% (0.52) | 19.9% (0.52) | 22.0% (0.49) |
| 5 | -1.1% (-0.02) | 3.4% (-0.07) | 0.3% (-0.02) | -0.1% (-0.02) |
| 6 | -1.0% (-0.02) | 1.0% (0.09) | -0.5% (-0.01) | -0.9% (-0.01) |
| 7 | -2.3% (-0.04) | -2.7% (-0.04) | -2.9% (-0.03) | -1.6% (-0.06) |
| 8 | -1.3% (-0.02) | 1.4% (-0.05) | -0.6% (-0.03) | -1.1% (-0.03) |
| 9 | -1.4% (-0.02) | -1.4% (-0.02) | -1.0% (-0.04) | -0.8% (-0.04) |
| 10 | -6.9% (-0.17) | -9.1% (-0.13) | -8.8% (-0.14) | -7.2% (-0.17) |
| 11 | -8.0% (-0.15) | -6.8% (-0.17) | -7.4% (-0.17) | -7.8% (-0.16) |
| 12 | -1.9% (-0.03) | -2.7% (-0.01) | -2.1% (-0.02) | -1.9% (-0.03) |
| 13 | -8.8% (-0.17) | -7.1% (-0.18) | -8.7% (-0.17) | -8.5% (-0.17) |
| 14 | -2.1% (-0.04) | -4.3% (-0.05) | -2.3% (-0.04) | -3.2% (-0.03) |
