# Supplementary material for: RESOLUTE PET/MRI Attenuation Correction for O-(2-18F-fluoroethyl)-L-tyrosine (FET) in Brain Tumor Patients with Metal Implants
Source: Front Neurosci. 2017 Aug 11;11:453. doi: 10.3389/fnins.2017.00453 (PMC5554515; doi:10.3389/fnins.2017.00453)
Supplement: Supplementary file 8 [file Presentation1.PDF]

Supplementary Material

RESOLUTE PET/MRI attenuation correction for O-(2-18F-fluoroethyl)-L-tyrosine (FET) in brain tumor patients with metal implants

Claes N. Ladefoged, Flemming L. Andersen, Andreas Kjær, Liselotte Højgaard, and Ian Law.

Department of Clinical Physiology, Nuclear Medicine and PET, Rigshospitalet, University of Copenhagen, Denmark

*** Correspondence:** Flemming Littrup Andersen: flemming.andersen@regionh.dk

1 Supplementary Data

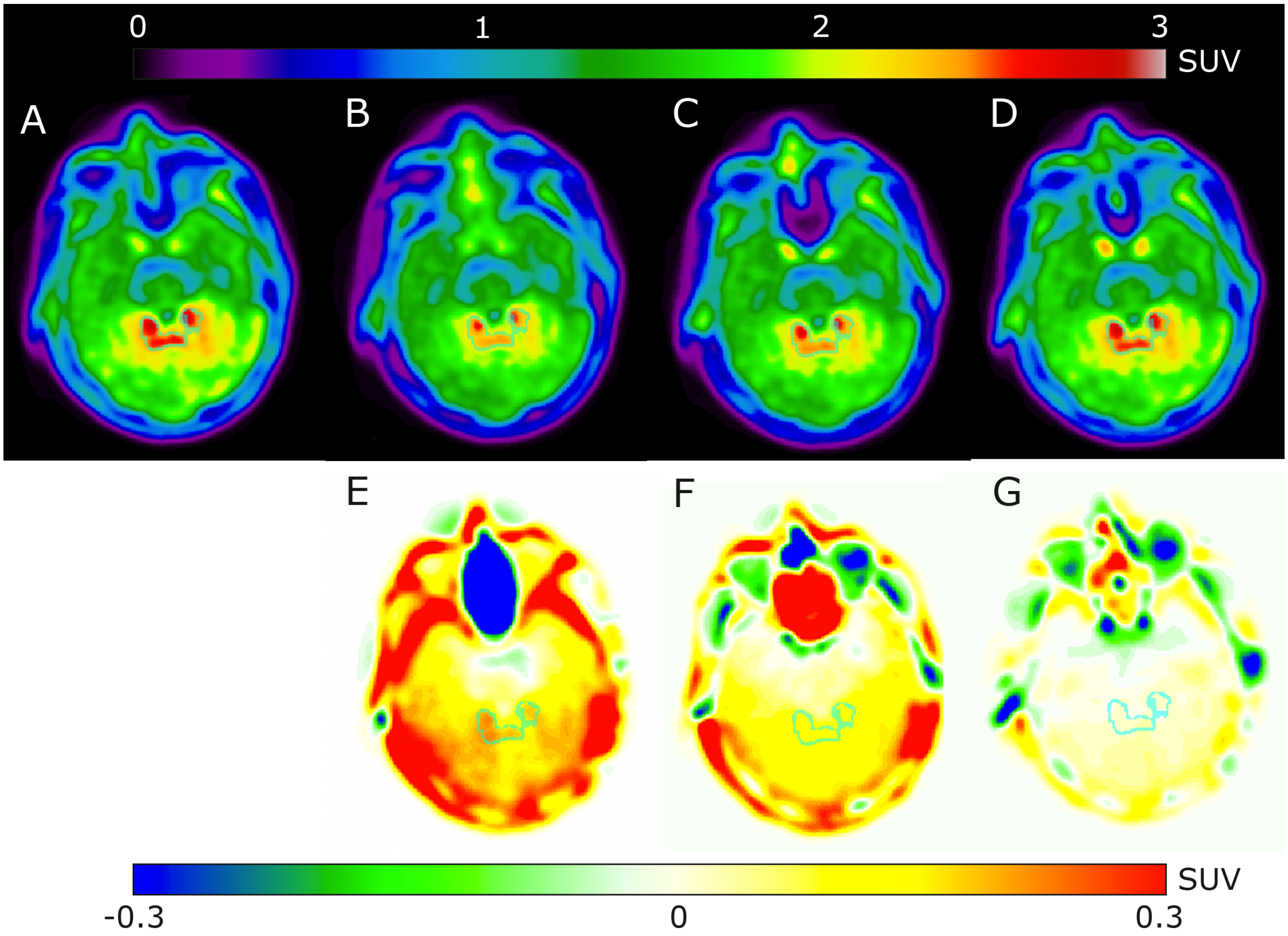

Supplementary Figure 1: Example of the attenuation corrected PET images for a single patient using the reference CT (A), Dixon (B), UTE (C) and RESOLUTE (D). The difference to the reference is shown for Dixon (E), UTE (F) and RESOLUTE (G). The tumor, as segmented on PET with CT-AC, is outlined in cyan in all images.
