# Supplementary material for: RESOLUTE PET/MRI Attenuation Correction for O-(2-18F-fluoroethyl)-L-tyrosine (FET) in Brain Tumor Patients with Metal Implants
Source: Front Neurosci. 2017 Aug 11;11:453. doi: 10.3389/fnins.2017.00453 (PMC5554515; doi:10.3389/fnins.2017.00453)
Supplement: Supplementary file 9 [file Presentation2.PDF]

Supplementary Material

RESOLUTE PET/MRI attenuation correction for O-(2-18F-fluoroethyl)-L-tyrosine (FET) in brain tumor patients with metal implants

Claes N. Ladefoged, Flemming L. Andersen, Andreas Kjær, Liselotte Højgaard, and Ian Law.

Department of Clinical Physiology, Nuclear Medicine and PET, Rigshospitalet, University of Copenhagen, Denmark

*** Correspondence:** Flemming Littrup Andersen: flemming.andersen@regionh.dk

1 Supplementary Data

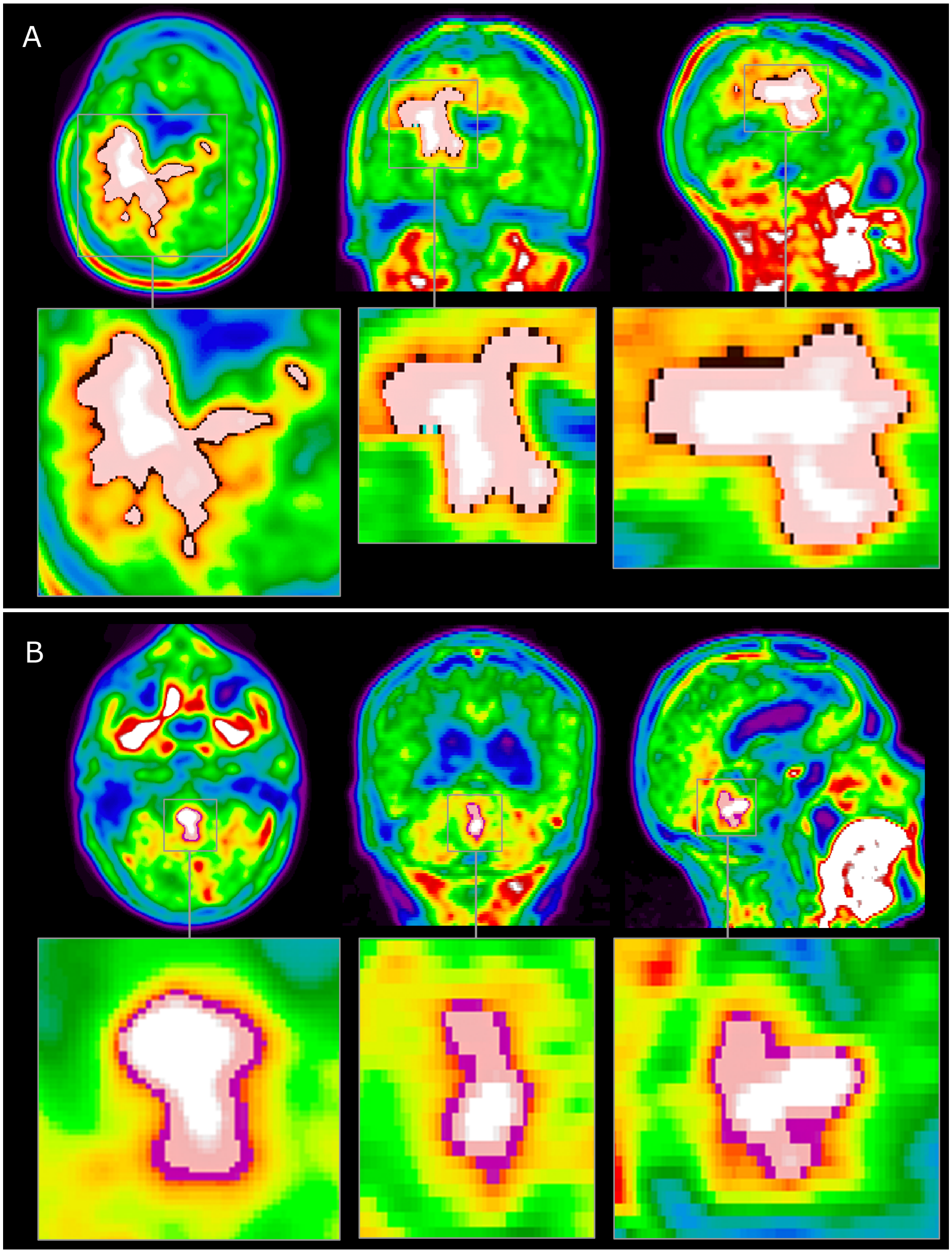

Supplementary Figure 2: The 2 patients with the largest biological tumor volume (BTV) differences using attenuation correction with RESOLUTE compared to CT. The white and pink areas show the tumor segmented with both. (A) An irregular anaplastic oligodendroglioma (WHO III) with diffuse boundaries. The black border shows the voxels overestimated by RESOLUTE with a BTV increase from 40.0 to 47.9 mL (20 %). (B) A glioblastoma (WHO IV) in the Vermis is underestimated by RESOLUTE (purple voxels) with a BTV decrease from 6.7 mL to 4.0 mL (40%). This may be caused by relative differences in activity in the background region. The difference would not have changed the reading of the images in relation to the clinical questions asked.
