# Supplementary material for: RESOLUTE PET/MRI Attenuation Correction for O-(2-18F-fluoroethyl)-L-tyrosine (FET) in Brain Tumor Patients with Metal Implants
Source: Front Neurosci. 2017 Aug 11;11:453. doi: 10.3389/fnins.2017.00453 (PMC5554515; doi:10.3389/fnins.2017.00453)
Supplement: Supplementary file 10 [file Presentation3.PDF]

Supplementary Material

RESOLUTE PET/MRI attenuation correction for O-(2-18F-fluoroethyl)-L-tyrosine (FET) in brain tumor patients with metal implants

Claes N. Ladefoged, Flemming L. Andersen, Andreas Kjær, Liselotte Højgaard, and Ian Law.

Department of Clinical Physiology, Nuclear Medicine and PET, Rigshospitalet,

University of Copenhagen, Denmark

*** Correspondence:** Flemming Littrup Andersen: flemming.andersen@regionh.dk

1 Supplementary Data

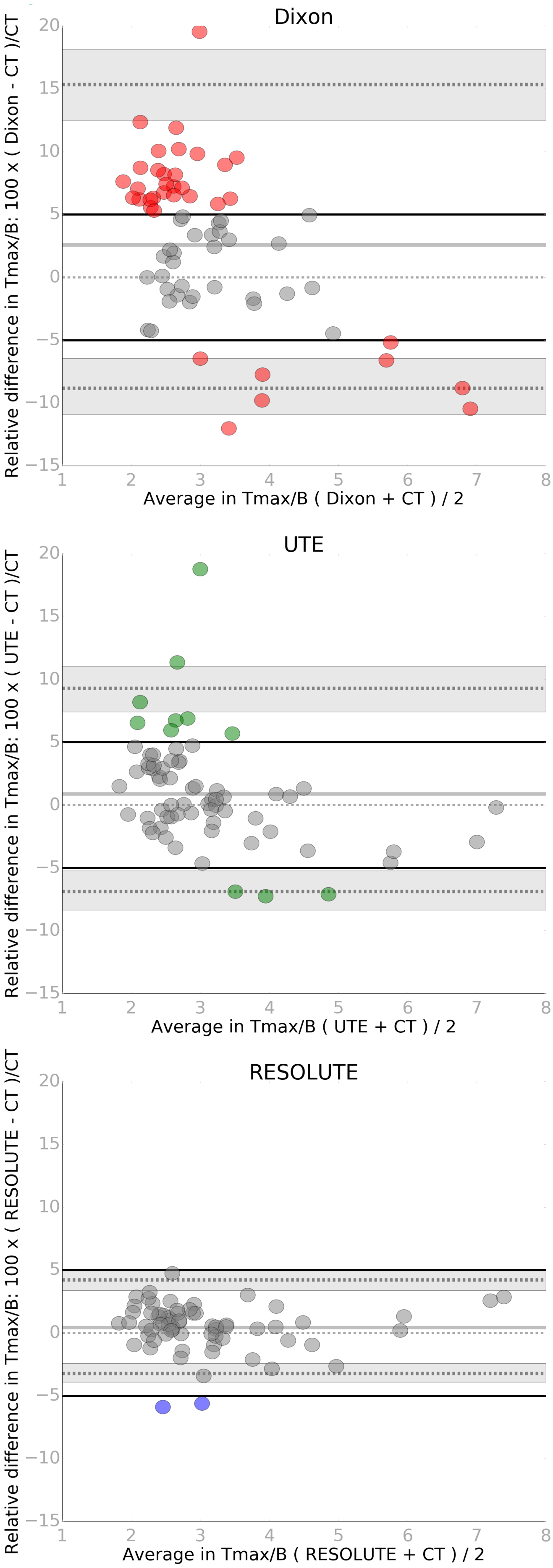

Supplementary Figure 3: Bland-Altman plots for T_{MAX}/B relative to CT-AC for each MR-AC method. The black lines indicate the acceptance criteria of T_{MAX}/B of 5% (±0.1 omitted here for illustrative purposes). Points that exceed the criteria have been colored. The solid gray lines indicate the mean value. The dotted gray lines indicate the lower and upper 95% limits of agreement. The shaded gray areas indicate the 95% confidence interval for the limits of agreement.
