# Supplementary material for: RESOLUTE PET/MRI Attenuation Correction for O-(2-18F-fluoroethyl)-L-tyrosine (FET) in Brain Tumor Patients with Metal Implants
Source: Front Neurosci. 2017 Aug 11;11:453. doi: 10.3389/fnins.2017.00453 (PMC5554515; doi:10.3389/fnins.2017.00453)
Supplement: Supplementary file 11 [file Presentation4.PDF]

Supplementary Material

RESOLUTE PET/MRI attenuation correction for O-(2-18F-fluoroethyl)-L-tyrosine (FET) in brain tumor patients with metal implants

Claes N. Ladefoged, Flemming L. Andersen, Andreas Kjær, Liselotte Højgaard, and Ian Law.

Department of Clinical Physiology, Nuclear Medicine and PET, Rigshospitalet, University of Copenhagen, Denmark

*** Correspondence:** Flemming Littrup Andersen: flemming.andersen@regionh.dk

1 Supplementary Data

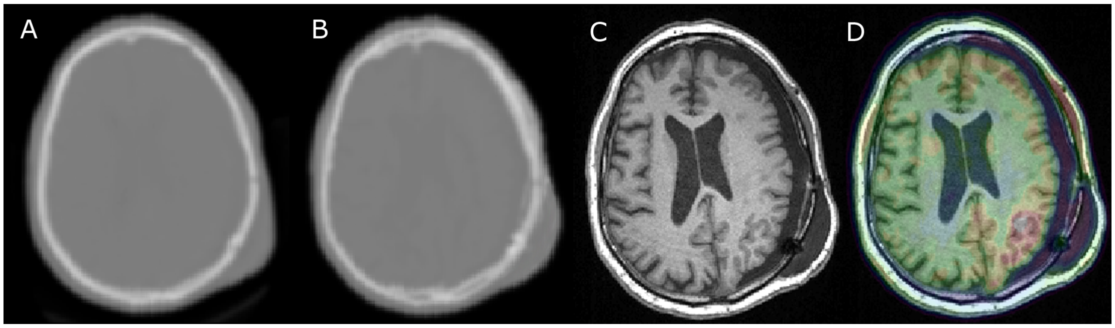

Supplementary Figure 4: An example of a challenging case with 12 mm thick postoperative soft tissue swelling at the resection site in the parietal region 21 days postoperative after resection of glioblastoma (WHO IV) showing good correspondence between attenuation correction using CT or RESOLUTE. (A) CT-AC, (B) RESOLUTE, (C) T1w MPRAGE, (D) FET-PET_{CT} fused onto MRI.
