# Supplementary material for: RESOLUTE PET/MRI Attenuation Correction for O-(2-18F-fluoroethyl)-L-tyrosine (FET) in Brain Tumor Patients with Metal Implants
Source: Front Neurosci. 2017 Aug 11;11:453. doi: 10.3389/fnins.2017.00453 (PMC5554515; doi:10.3389/fnins.2017.00453)
Supplement: Supplementary file 12 [file Presentation5.PDF]

Supplementary Material

RESOLUTE PET/MRI attenuation correction for O-(2-18F-fluoroethyl)-L-tyrosine (FET) in brain tumor patients with metal implants

Claes N. Ladefoged, Flemming L. Andersen, Andreas Kjær, Liselotte Højgaard, and Ian Law.

Department of Clinical Physiology, Nuclear Medicine and PET, Rigshospitalet, University of Copenhagen, Denmark

*** Correspondence:** Flemming Littrup Andersen: flemming.andersen@regionh.dk

1 Supplementary Data

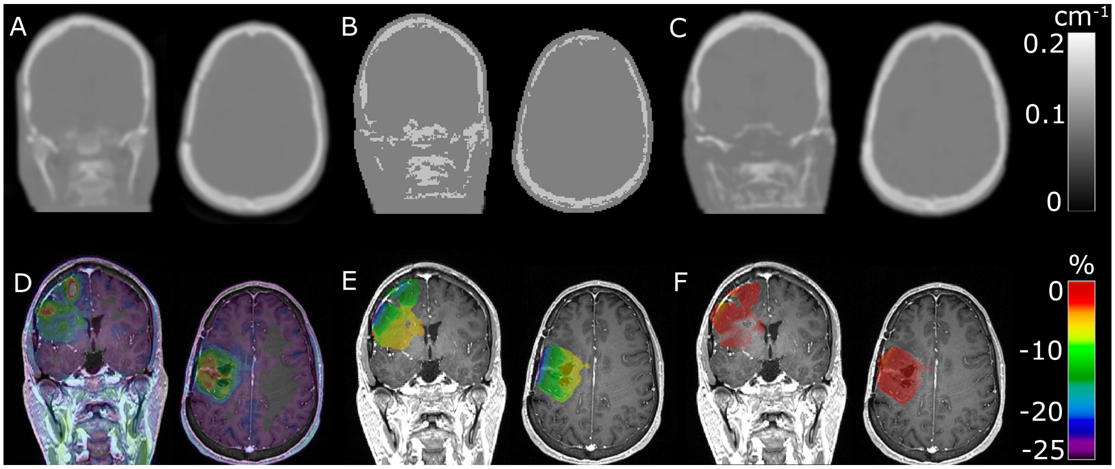

Supplementary Figure 5: Within tumor attenuation correction method dependent gradient effects. Top row: attenuation maps from (A) CT, (B) UTE, (C) RESOLUTE. Bottom row: (D) FET-PET$_{CT}$ fused with MRI, (E, F) Within tumor relative FET PET difference images from UTE and RESOLUTE, respectively, fused with MRI showing a marked 20% radial gradient for UTE and only a minor 2% gradient for RESOLUTE.
